# Endocarditis due to *Rhodotorula mucilaginosa* in a kidney transplanted patient: case report and review of medical literature

**DOI:** 10.1099/jmmcr.0.005119

**Published:** 2017-11-02

**Authors:** Andrea Maria Cabral, Suzimar da Siveira Rioja, Fabio Brito-Santos, Juliana Ribeiro Peres da Silva, Maria Luíza MacDowell, Marcia S. C. Melhem, Ana Luíza Mattos-Guaraldi, Raphael Hirata Junior, Paulo Vieira Damasco

**Affiliations:** ^1^​ Faculdade de Ciências Médicas - Universidade do Estado do Rio de Janeiro - UERJ, Rio de Janeiro, Brazil; ^2^​ Laboratório de Micologia do Instituto Nacional de Infectologia (INI), Evandro Chagas, FIOCRUZ, Rio de Janeiro, Brazil; ^3^​ Laboratório Central-Hospital Universitário Pedro Ernesto-UERJ, Rio de Janeiro, Brazil; ^4^​ Universidade Federal do Estado do Rio de Janeiro -UNIRIO, Rio de Janeiro, Brazil; ^5^​ Instituto Adolfo Lutz, São Paulo, Brazil

**Keywords:** *Rhodotorula mucilaginosa*, infective endocarditis, immunocompromised, kidney transplantation

## Abstract

**Introduction.** Endocarditis caused by yeasts is currently an emerging cause of infective endocarditis and, when accompanied byfever of unknown origin, is more severe since interferes with proper diagnosis and endocarditis treatment.

**Case presentation.** The Rio de Janeiro Infective Endocarditis Study Group reports a case of infectious endocarditis (IE) with negative blood cultures in a 45-year-old white female resident in Rio de Janeiro, Brazil, previously submitted to kidney transplantation. After diagnosis and intervention, the valve culture revealed *Rhodotorula mucilaginosa*. The clinical aspects and overview of endocarditis caused by *Rhodotorula* spp. demonstrated that *R. muscilaginosa* have been isolated from the last IE cases from kidney transplanted patients.

**Conclusion.** Though most of the patients (in literature) recovered well from endocarditis caused by *Rhodotorula* spp., physicians must be aware for diagnosis of fungemia and fungal treatment in kidney transplanted patients suffering of fever of unknown origin in the modern immunosuppressive treatment.

## Abbreviations

CAIE, Community-Acquired infectious endocarditis; CAPES, Coordenação de Aperfeiçoamento de Pessoal de Nível Superior, from the Ministry of Culture/Brazil; CNPq, Conselho Nacional de Desenvolvimento Científico e Tecnológico, from the Ministry of Technology/Brazil; FAPERJ, Fundação de Amparo a Pesquisa do Estado do Rio de Janeiro; FE, fungal endocarditis; FUO, fever of unknown origin; HAIE, Healthcare-Associated infectious endocarditis; HUPE, Pedro Ernesto University Hospital of State University of Rio de Janeiro; IE, infectious endocarditis; ITS, nuclear ribosomal internal transcribed spacer (ITS) region; LILACS, Latin America Scientific Literature; MALDI-TOF, matrix assisted laser desorption-time of flight; MEDLINE, U.S. Library of Medicine; PubMed, WEB version of MEDLINE; SciELO, Scientific Electronic Library Online; UERJ, Universidade do Estado do Rio de Janeiro/State University of Rio de Janeiro.

## Introduction

Fungal endocarditis (FE) is currently an emerging cause of infective endocarditis (IE). Although the most frequently fungal pathogens isolated from FE are *Candida* spp., there are other fungal agents including *Aspergillus* spp., and *Histoplasma capsulatum* [1–3]. *Rhodotorula* spp. is a basidiomycetous yeast, considered a member of the *Cryptococcaceae* family, and was previously described as a rare etiological agent in culture negative infective endocarditis [4, 5].

Infective endocarditis (IE) is an infection located in the endocardial valve(s), and according to the acquisition of organisms involved, is classified as Community-Acquired (CAIE) or Healthcare-Associated (HAIE). The estimated annual incidence of IE ranges from 3 to 9 per 100 000 in developed countries [6–8].

Even though the access to a microbiology laboratory and epidemiological data of IE in developing countries is scarce in medical literature, our group has shown that in Brazil, HAIE is more prevalent than CAIE in our cohort of cases in Rio de Janeiro. Our group has reported that *Staphylococcus aureus* was the most frequent (30 %) followed by *Enterococcus faecalis* (26.7 %) microorganisms isolated from positive blood cultures [9].

We hereby report a case of infective endocarditis due to *Rhodotorula mucilaginosa* in a kidney transplanted patient, who was admitted to our teaching hospital with fever of unknown origin (FUO). Thereafter an overview of cases of IE due to *Rhodotorula* spp. in English, Spanish and Portuguese literature since 1960 was done, and we have reported the 10th case.

## Case report

**Fig. 1.**
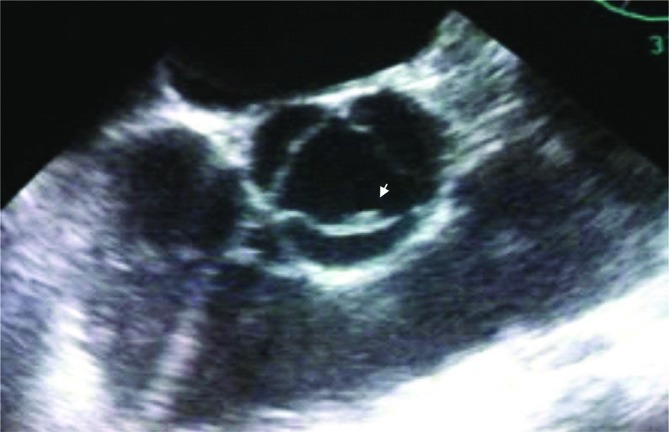
Infective endocarditis (IE) due to *Rhodotorula mucilaginosa.* A transesophageal echocardiogram showed a 0.3 cm thickening in the ventricular side of aortic valve (arrow).

A 45-year old woman, with a history of deceased-donor kidney transplant in 2004, was admitted at HUPE in April 2012, for investigation of FUO. Three days after the admission, she developed daily peaks of fever varying from 38.0 to 39.3 °C, with intermittent fever pattern. Her complaints were fever and abdominal pain for 3 weeks prior to admission. She was under a combined imunossupressive therapy of Azathioprine, Sirolimus and Prednisone. Six peripheral blood culture sets were drawn on admission and incubated in BacT/Alert standard aerobic, after a 2 week investigation for the cause of FUO, all the blood culture sets were negative. In the beginning, the transthoracic echocardiography and radiologic studies were all inconclusive. After insisting on searching IE, a transesophageal echocardiography showed a heterogeneous mobile lesion adherent to the ventricular side of the aortic valve with 0.30 cm thickening and mild ventricular regurgitation (Fig. 1). Empirical antibiotic therapy was initiated with vancomycin and ciprofloxacin but failed to reduce the fever, which persisted for the following 2 weeks. The patient was then submitted to cardiac surgery, in which the aortic valve was found to be deformed by the vegetation. A fragment of the valve was sent to the microbiology laboratory for microbiological culture and DNA extraction for further search of micro-organisms involved in blood culture-negative organisms. After maceration of the valve in sterilised phosphate buffered saline, aliquots (10 µl) of the suspension were seeded into Thioglycollate Broth and in anaerobic supplemented blood agar base, and incubated in both aerobic and anaerobic conditions at 37 °C. The Gram-stain of the suspension demonstrated yeast cells, and a 10 µl aliquot was also seeded in Sabouraud medium containing 10 mg ml^−1^ chloramphenicol, incubated at room (±23 °C) and 37 °C temperatures. The yeast grew in pure culture only after 72 h of incubation at room temperature. Sabouraud tubes also incubated at 37 °C demonstrated no growth. The yeast was plated in Blood Agar Base and incubated at room temperature for 72 h, and revealed dark-red colonies, with microscopic view of budding yeast cells, and positive reaction on Gram-staining (Gram-positive). The yeast was phenotypically characterised as *Rhodotorulla* spp. MALDI-TOF analysis identified the yeast as *Rhodotorula mucilaginosa*. PCR targeting the ITS region was performed and a fragment around 739 pb was observed. Sequences generated after automated sequencing presented 99 % homology with *Rhodotorula mucilaginosa*. The sequence was deposited at NCBI (KY113079). E-test (bioMérieux) showed susceptibility to amphotericin B (Amp B, 0.25 mg ml^−1^), voriconazole (0.50 mg ml^−1^) and flucytosine (5-FC, 0.19 mg ml^−1^). Two resistance profiles were observed for fluconazole (>256 mg ml^−1^) and for itraconazole (>32 mg ml^−1^). The patient was discharged after a 40 day therapy treatment with liposomal amphotericin B.

## Discussion

The prevalence of IE depends on the underlying heart disease, including structural congenital heart disease, rheumatic fever, degenerative heart disease, intravenous drug addiction, reconstructive cardiac surgery, pacemakers and implantable cardioverter defibrillator, the prolonged use of intravenous catheters, immunocompromised and diabetic patients. The institutions have patients undergoing hemodialysis therapy and immunocompromised patients receiving cytostatic cancer chemotherapy have a higher prevalence of HAIE [6–10].

*Rhodotorula* spp. has been isolated from different sites including skin, nails, conjunctiva, as well as from respiratory and gastrointestinal tracts [11, 12]. Although *Rhodotorula spp*. has a low prevalence in fungal endocarditis (FE), compared to *Candida* spp., *Aspergillus* spp. and *Histoplasma capsulatum*, the infective endocarditis team or internal medical physician should consider this fungus. *Rhodotorula* ssp. is a high risk for IE in a host with central venous catheter or immunosuppression [5, 11]. A search of MEDLINE, PubMed, Scielo and LILACS for endocarditis caused by *Rhodotorula* using the terms: ‘fungal endocarditis’, ‘fungus endocarditis’, ‘Endocarditis due to *Rhodotorula*’, ‘Infective Endocarditis caused by *Rhodotorula*’, in our overview, this case report is the 10th (Table 1) case of IE due to *Rhodotorula* since 1960 [1, 4, 13–19]. Amongst the genus, *Rhodotorula mucilaginosa* seems to be the most pathogenic species, and was responsible for 54.5 % cases of endocarditis, including in the last two described cases, occurring in kidney transplanted patients (Table 1).

**Table 1.** Summary of the case reports of infective endocarditis (IE) due to *Rhodotorula spp.* found in the literature (*n*=9)

Year	Country/Reference	Age/Sex	Risk factors	Valve/type*	Species	Blood culture	Valve culture	Antifungal treatment†	Outcome
1960	USA/1	47/F	Mitral and aortic stenosis from rheumatic fever, dental procedure, indwelling catheter.	Ao/Native	ns	+	+	None	Deceased
1962	USA/13	56/M	Diabetes, rheumatic fever, prolonged urinary catheter, decubitus ulcer	ns	ns	+	np	Amp B	Recovered
1969	USA/14	39/M	Dental procedure, prolonged urinary catheter, decubitus ulcer	Mi/native	ns	+	np	Amp B	Recovered
1975	Israel/15	7/M	Recurrent tonsillitis, tonsillectomy	Mi/Ao/native	*R. pilimanae*	+	np	Flucy	Recovered
2003	Switzerland/16	53/M	Prosthetic valve, antibiotic use, endocarditis	Ao/Prosth.	*R. mucilaginosa*	–	+	Amp B+Itrac	Recovered
2005	Switzerland/17	56/M	Cardiac transplant recipient	Left Atrium appendice	*R. glutinis*	+	+	Lipos Amp B	Recovered
2005	Brazil/18	10/F	Central venous catheter	Right Atrium appendice	*R. mucilaginosa*	–	n.p.	Amp B+Flucy+Rifampicin	Recovered
2011	Brazil/19	58/M	Coronary stent	Ao/Native	*R. mucilaginosa*	np*	np	Amp B	Recovered
2014	USA/4	54/F	Diabetes, kidney transplant	Ao/Bioprosth.	*R. mucilaginosa*	+	+	Lipos AmpB	Recovered
2017	Brazil‡	45/F	Kidney transplant	Ao/Prosth.	*R. mucilaginosa*	–	+	Lipos AmpB	Recovered

*Valve/Type: Mi, Mitral; Ao, Aortic; Prosth, Prosthetic; Bioprosth, Bioprosthetic; ns, Not specified; np, Not performed.

†Antifungal therapy: AmpB, Amphotericin B; Flucy, Flucytosine; Amp B+Itrac, Amphotericin B+Itracoconazole; Lipos AmpB, Liposomal Amphotericin B.

‡Case presented in this report.

*Rhodotorula* spp. has been reported in cases of fungemia, sepsis, meningitis, ventriculitis, peritonitis, keratitis, endophtalmitis, dacryocystitis, pneumonia, IE and more recently has been considered as an emerging pathogen [11, 12, 16–18]. The immunocompromised populations are at greatest risk of fungus infection. There are some reports of *Rhodotorula* infections in cancer patients with solid tumors, lymphoproliferative disease, HIV, diabetes mellitus and chronic renal failure [5, 11]. In Rio de Janeiro, IE due to *R. mucilaginosa* was reported in a 45-year old kidney transplanted female patient where we found a vegetation of aortic valve even though blood cultures were negative. The frequency of *Rhodotorula* infections is reported in both genders since the first description in 1960, and with a mean of 41 years (Table 1).

In our overview, the left valve of the heart was more frequently implicated with *Rhodotorula* IE than the right valve. Among the nine patients previously reported in literature five of them involved aortic valve and only 30 % was related in solid organ transplant (Table 1). *Rhodotorula* infective endocarditis was reported associated with the widespread use of broad spectrum antibiotics and steroids in many chronic diseases [14]. Notwithstanding, it is possible that *Rhodotorula* spp. may be implicated with native valve, right sided heart infections and infective endocarditis in children and imumunocompetent patients [15, 19].

The yeast identification is ideal for the management of fungal endocarditis (FE) [3, 4]. The widespread prophylaxis and the empirical treatment of fungemia with triazole antifungal agents may also allow the emergence of specifically resistant fungi, including *Rhodotorula* species, due to its natural resistance to fluconazole and echinocandins [2, 5]. In the first report case of *Rhodotorula* IE, the patient died due to the absence of administration of anti-fungal treatment [1]. In our case, the patient was discharged after 40 days of treatment with liposomal amphotericin B and valve surgery.

One aspect that calls attention is the emergent isolation of *R. mucilaginosa* from patients accompanied after kidney transplantation, and in one patient after heart transplantation, *R. glutinis* was isolated (Table 1). When positivity of blood cultures is taken into consideration, four (36.7 %) case reports had negative blood cultures for infective endocarditis. Three cases according our overview occurred in Brazil and one in Switzerland (Table 1). Thus, *Rhodotorula* spp. can be involved in negative blood culture endocarditis, and the culture of the valve can provide the isolation of the microorganism, as recently stated [20]. Also, attention to incubation of culture media (at room temperature, ~25 °C) is also necessary for a proper growth and identification of *R. mucilaginosa*.

*Rhodotorula* spp. is an emerging opportunistic pathogen, particularly in immunocompromised patients. We need to improve medical microbiologic laboratory testing for fungemia diagnosis in renal transplantation population in the modern immunosuppressive treatment era.
